# “There’s a huge benefit just to know that someone cares:” a qualitative examination of rural veterans’ experiences with TelePain

**DOI:** 10.1186/s12913-021-07133-5

**Published:** 2021-10-16

**Authors:** Molly Silvestrini, Jess Indresano, Steven B. Zeliadt, Jessica A. Chen

**Affiliations:** 1 Department of Veterans Affairs Puget Sound Health Care System, Seattle, USA; 2 Department of Psychiatry & Behavioral Sciences, University of Washington, Seattle, USA; 3 Department of Health Services, University of Washington, Seattle, USA

**Keywords:** Chronic pain, Veterans, Telehealth, Rural, Qualitative

## Abstract

**Background:**

Veterans in the United States are at an increased risk of chronic pain and have higher reported pain prevalence and severity than nonveterans. This qualitative study aims to examine veterans’ perspectives on the acceptability of receiving pain care through TelePain, a telehealth program implemented by the Veterans Health Administration (VA) that offers specialty pain care to rural veterans in their homes or in a video conferencing room at a nearby outpatient clinic.

**Methods:**

The VA electronic health record was used to identify patients who were referred to TelePain from rural clinics located in Washington, Oregon, and Alaska between 12/01/2019 and 03/31/2020. The study team completed 16 semi-structured interviews with rural veterans about their experiences with TelePain. After interview transcripts were recorded digitally and transcribed, Atlas.ti was used to organize data and facilitate qualitative coding. Interview transcripts were analyzed using thematic analysis.

**Results:**

Veterans reported general satisfaction with receiving pain care through telehealth and valued having supportive, knowledgeable providers who provided useful information and resources. In addition, veterans appreciated the convenience of telehealth. Barriers to care included problems with program follow-up, negative perceptions of mental health care for pain, and preference for in-person care. Although some patients suggested that telehealth audio and video could be improved, most patients did not have any significant problems with telehealth technology.

**Conclusions:**

In this sample of rural veterans who used TelePain, many reported satisfaction with the program and positive experiences with providers. Targets for quality improvement include streamlining the program’s referral and scheduling process and improving patient motivation to engage in psychological pain treatments. Results indicate that delivering pain services over telehealth is an acceptable modality for this patient population.

**Supplementary Information:**

The online version contains supplementary material available at 10.1186/s12913-021-07133-5.

## Background

Chronic pain is a persistent condition and public health issue that disproportionately affects the veteran population [1]. Untreated chronic pain can contribute to depression, anxiety, disability, poor sleep, and decreased quality of life [2]. Veterans in the United States are at an increased risk of chronic pain and have higher reported pain prevalence and severity than nonveterans [1]. According to Department of Veterans Affairs’ Office of Rural Health (ORH), 4.7 million veterans live in rural communities across the U.S. and face challenges such as long transportation distances to access care, lack of internet access at home, and fewer health care providers and nurses in their communities [3]. Veterans who live in rural areas are less likely to receive comprehensive pain care [4], are prescribed over 30 % more opioids than their urban counterparts [5], and report lower use of self-management interventions for pain [6], suggesting a high need for quality pain care in rural areas.

Previous research suggests that Veterans Health Administration (VA) telehealth programs, which often involve a computer-to-computer (or video teleconference technology-to-home-based computer) clinical interaction [7], may provide valuable care to veterans living in rural areas. An evaluation of the first VA home-based telemental pilot program found that rural veterans reported high levels of satisfaction and perceived safety with home-based telemental health, as well as fewer no-show appointments when compared to clinic-based telemental health [7]. In an implementation project of VA Video to Home (VTH) designed to deliver evidence-based psychotherapies to underserved rural veterans, patients reported that VTH addressed barriers to care such as distance to a VA clinic and availability of transportation, parking, work schedules, and health conditions that made travel less feasible [8]. Less is known about the acceptability of pain care over telehealth. One study used qualitative methods to assess women veterans’ acceptability of telehealth care for chronic pain and comorbid depression and/or posttraumatic stress disorder and found clinic-to-clinic telehealth to be just as acceptable as in-person care [9].

As a response to the need for chronic pain care among rural veterans, the VA Veterans Integrated Service Network (VISN) 20 (or the VA Northwest Health Network) implemented TelePain, a telehealth program for improving access to pain treatment options in rural Alaska, Washington, and Oregon [10], particularly behavioral treatment options that are not widely available outside of specialized pain rehabilitation programs [11]. TelePain delivers primarily non-pharmacological chronic pain care, such as psychological therapies (e.g., Cognitive Behavioral Therapy for Chronic Pain) [12] and physical movement classes using video telehealth. Additional details about TelePain program development, staffing, and treatment services have been described in previous literature [13]. Understanding patient acceptability and treatment satisfaction are critical for evaluating the value of delivering pain care over telehealth and to inform future implementation efforts. The present study sampled a population of patients with chronic pain who participated in the regional (i.e., multi-state) TelePain program. To evaluate patient satisfaction with the delivery of chronic pain care through telehealth, this study used qualitative, semi-structured interviews to examine 16 rural veterans’ experiences with TelePain.

## Methods

### Setting

The VA VISN 20 TelePain program is a hub-and-spoke telehealth program [14] wherein pain specialists affiliated with a large academic medical center (VA Puget Sound Health Care System in the Seattle-Tacoma, WA metropolitan area) deliver cognitive behavioral therapy, complementary health, and physical movement classes for chronic pain to rural veterans affiliated with clinical spokes, local community-based outpatient clinics (CBOCs) focused on primary care practice [13]. TelePain specialists are physically located in a single VA medical center at one of two divisions (Seattle, WA or Tacoma, WA). TelePain is built on the model of interdisciplinary, team-based pain management [15]. TelePain’s outpatient rehabilitation-focused services are based on a biopsychosocial model of chronic pain. Treatment offerings include pain education (group classes), evidence-based psychotherapies (individual and group), physical therapy-based movement classes (individual and group), and opioid safety consultations (individual). The goal of TelePain is to reduce reliance on opioids for pain management and to increase access to safer, more comprehensive non-pharmacological pain treatment options among rural veterans [13].

TelePain’s video telehealth services are delivered both clinic-to-clinic (from specialist-staffed medical center to CBOC) and direct-to-home (from specialist-staffed medical center to patients’ homes using internet-enabled devices). Patients are referred by their primary care providers or occasionally by their mental health or medical specialists for the purpose of engaging in non-pharmacological treatments for chronic pain. When referring patients, providers are asked to include a recent evaluation of suicide risk so that the team may triage any patients at acute risk of self-harm to the telemental health team; failure to include suicide risk information results in the referral being sent back to the provider. Following triage, patients are scheduled for an individual or group pain education session to introduce the biopsychosocial model of chronic pain care followed by a clinic intake. During the intake, providers and patients develop an individualized treatment plan.

TelePain is comprised of 16 full-time equivalent employees, including a program manager, eight behavioral health providers (psychologists, psychiatrists, and social workers), one physical therapist, and six administrative support staff, including medical support assistants for scheduling and telehealth clinical technicians for technical support. The effort of these staff is primarily devoted to the TelePain clinic, though all staff are also affiliated with a local pain rehabilitation program that is accredited by the Commission on Accreditation of Rehabilitation Facilities.

### Participants

Using the VA electronic health record, we identified patients who were referred to TelePain from rural clinics located in Washington, Oregon, and Alaska between 12/01/2019 and 03/31/2020. In order to facilitate and examine diverse experiences accessing the TelePain program, we recruited both veterans who had participated in the TelePain program (i.e., completed the referral, *n* = 20) and some who had not (*n* = 11). Veterans were sent a recruitment letter by mail indicating that project staff would call them within two weeks if they did not opt out. Staff called each potential participant up to three times to invite them to participate in an interview about TelePain. This project was categorized as quality improvement by the VA, and therefore did not require approval from the local institutional review board. Because this quality improvement project did not include funding for participant payments, participants were not compensated for the interviews.

### Data collection

Three members of the study evaluation team (a PhD-level clinical psychologist and two health science research specialists, MPH and BA) developed a semi-structured interview guide designed to elicit veterans’ experiences with TelePain. Between March 2020 and June 2020, two health science specialists trained in qualitative research conducted semi-structured phone interviews with 16 veterans enrolled at rural VA clinics. Participants gave informed verbal consent to be audio recorded before each interview. Interviews were professionally transcribed. Each interview lasted approximately 20–30 min. During the interviews, TelePain was evaluated across the following domains: connecting with TelePain, treatment outcomes, and usability of TelePain technology. Participants were asked about their experiences with TelePain, including questions about why they did or did not participate in the program, their perceptions of TelePain providers, barriers they faced accessing the program, suggestions for improvement, and experiences using telehealth technology to access pain care.

### Data analysis

Atlas.ti (Version 8, Berlin, Scientific Software Development) was used to organize data and facilitate qualitative coding. First, two members of the study team independently reviewed two transcripts in order to develop codes that would reflect and categorize veterans’ experiences with TelePain, as well as capture data elicited by questions from the interview guide. During this process, the entire team met to discuss and refine a codebook for analysis and develop a process for resolving differences in coding through discussion and consensus. Next, the two analysts coded two of the same transcripts using the codebook, then met to discuss accuracy of codes and resolve differences. Once all discrepancies were resolved and the codebook was finalized, the two analysts divided and coded the remaining transcripts independently. Once coded, the interview transcripts were analyzed using thematic analysis, a qualitative method for classifying, analyzing, and describing patterns and themes within data and across cases [16, 17]. Thematic analysis can include a range of methods, from techniques that “prioritize coding accuracy and reliability” to reflexive approaches that “emphasize the inescapable subjectivity of data interpretation” [17]. Our approach utilized coding accuracy and reliability to examine patterns and themes within the data. Thematic analysis was selected as our analytic process because it is an extremely flexible approach that provides a thorough and comprehensive, yet intricate account of data [18, 19]. Themes identified during this process were then discussed by the entire team for accuracy and significance.

## Results

Of the 33 potentially eligible veterans, 12 did not respond to recruitment calls and 5 declined to participate. Sixteen veterans completed interviews. Among our sample, 11 veterans had participated in TelePain and 5 had not. Participants were predominantly male (75 %), white (93.75 %), and ranged in age from 41 to 73 years old (mean of 60). This demographic data is based on retrospective chart review of patients’ electronic health records, which was completed only for patients that agreed to participate. The majority of participants (87.5 %) had > 1 chronic pain-related diagnosis in their medical record. 75 % of participants were diagnosed with joint pain and/or arthritic disorder, 50 % with back pain, 31 % with neuropathy, and 12.5 % each with fibromyalgia and headache. Our sample was similar to previous research describing a VA telehealth pilot program and the characteristics of Veterans with chronic pain needs who received telehealth services [13].

Although patients had unique and varied experiences with TelePain care, a majority of patients who had used TelePain reported positive experiences. Through thematic analysis, the team identified four salient themes: (1) Patients valued the support, information, and resources they received from TelePain providers; (2) TelePain is convenient and alleviates travel burden; (3) Barriers to TelePain participation include a perceived lack of program follow-up, negative perceptions of mental health pain care, and preference for in-person care; and (4) Patients did not have significant issues using telehealth technology.

### Theme 1: Patients Valued the Support, Information, and Resources They Received from TelePain Providers

A majority of veterans recounted positive and supportive interactions with their TelePain providers. Veterans described their providers as “*kind”* or “*nice,”* and recounted working together collaboratively as a team to address chronic pain. One patient said, *“[Provider] was willing to help, and I’m willing to learn. We’re working well together as a team*.” Another patient said, *“At first it was a little bit awkward, because I didn’t know what to expect. But the person I spoke to, she was very kind. I liked her.”* A third patient stated that they look forward to their TelePain visits because *“[Provider] is very relaxed, very easy going, and we get into some pretty thick conversations, so we’re able to meet the purpose of the contact and the benefits are good.”*

One patient appreciated the validation and emotional support they received from their TelePain provider. They said: *“A lot of people don’t understand, a lot of veterans are accused of drug seeking, and that’s not always true. There are people that do that, but it’s because their emotional pain is not dealt with. So, I think telehealth is good, you can see the person you’re talking to and you read their body language and you pick up the signals. And I think that’s a great asset for the VA as well as the veteran. There’s a huge benefit just to know that someone cares.”*

Patients also spoke of their providers as knowledgeable clinicians who provided helpful resources. One patient said: *“[Provider] is very knowledgeable. I think that’s a big deal for me. She has an idea of what you’re discussing. She can understand if you relate a feeling about something, she understands.”* Another patient reported that their TelePain provider “*seemed to have done his research*” prior to their visit and “*knew the case*.” Another patient commented that their TelePain provider gave them “*good, basic information*” and added: “*That’s what you need when you enter into a program like this, to understand what’s going on. It helped me to understand what’s going on*.”

Veterans also appreciated the way in which information was communicated by their providers; one patient said, “*I liked the fact that she took her time and explained and made sure you understood*.” Another stated: *“What it did, it gave me several resources to contact in case something was going on. So, I had these resources by the phone, I could just call and say, ‘this is going on.’ And before, I didn’t know I could do that.”*

### Theme 2: TelePain is Convenient and Alleviates Travel Burden

Many veterans appreciated the convenience remote visits offered and reported that telehealth helped alleviate their travel burden. One patient stated: *“I’m agoraphobic, so it’s much better for me to be able to do this at home rather than going to the clinic. So, I think that’s a huge benefit to a lot of veterans that are like myself.”* Another patient said: *“I think the thing I really liked about it was the fact that I was sitting in my own home and sipping on a cup of coffee through the meeting, it just was a very nice environment for me to be in for the meeting.”*

When asked what they liked about TelePain, one veteran answered, “*Just that it’s convenient. I can just lay here and talk to [Provider] and converse. I don’t have to go to [the medical center].*” Another patient said that participating in TelePain was easier than going to a VA clinic because “*You know, you don’t have to buy gas, you don’t have to travel, you don’t have to get a hotel somewhere, because I’m coming such a ways away, they usually get me a room*.”

Many of these patients mentioned that they would like to continue to use TelePain because of its convenience, rather than having to travel far distances to receive pain care. When asked if they would use TelePain again, one patient said: “*Oh, of course. It’s much easier than going clear to [the VA medical center]*.” Another patient replied, “*Yeah, probably. It’s easier for me than driving to [the VA] and back*.”

### Theme 3: Barriers to TelePain Participation Include a Perceived Lack of Program Follow-Up, Negative Perceptions of Mental Health Pain Care, and Preference for In-Person Care

Some patients who were referred to the program but did not participate reported that a perceived lack of follow-up from TelePain providers and scheduling staff caused problems when trying to make appointments. One veteran reported they were interested in participating but had not been able to due to trouble connecting with the program: “*I was trying to find my options to see what I could do…I guess they put a referral in and I’m still waiting to hear about starting. I haven’t heard anything.*” When asked to expand on this comment, the same patient said, “*I mean that’s really it. I can’t tell you because I haven’t even started the process. If they took the initiative, I might be able to*.”

One patient who was referred to the program reported that they never received any information about TelePain, including from their referring provider, and stated, “*I don’t think I’ve heard anything about this program at all*.” Another veteran who was referred reported issues knowing when their TelePain appointment was: “*I had a problem with that because a specific appointment wasn’t given to me, but they said they would be calling me within a week or so to let me know when the next meeting was going to be, but I haven’t received that call*.”

A few patients reported they had negative perceptions of participating in a program focused on non-pharmacological, mental health management of chronic pain. Some of these perceptions were due to negative or ineffective experiences seeing mental health providers in the past. One patient said: *“I don’t particularly like seeing psychiatrists. That’s just an opinion from years and years of dealing with this crap. I can keep talking about the pain all day, it’s not going to make it go away. I don’t want to keep doing more of that stuff if it’s not effective.”*

Another veteran who did participate in TelePain was initially hesitant because of their lack of experience with mental health pain providers. The patient said, *“I think the troubling part for me was I’ve never dealt with a psychologist before, I mean, really.”* When recounting their brief encounter with a psychologist in the past, the patient reported, *“He didn’t help me at all. It made me feel really uncomfortable.”* However, when the patient later met with a TelePain provider, these feelings changed: “*The more we talked, the less I felt like I was talking to a psychologist, I started feeling more like I was talking to a friend, a person.”*

In addition to perceived lack of program follow-up and negative perceptions of mental health pain care, a minority of patients expressed preferences for in-person care. One veteran who was referred to the program but did not participate said, “*I’m kind of old school, I like talking to my doctors face-to-face. I like looking in people’s eyes when they’re talking to me, that’s how I judge people*.” Another patient who did participate in the program commented: *“It’s more personable when you see a person face-to-face, they can look at you and you can look at them. You can read their face and their body language. It’s a whole different kind of communication.”*

### Theme 4: Patients Did Not Have Significant Issues Using Telehealth Technology

Although some patients suggested that TelePain technology could be improved, a majority of patients did not have any significant problems using telehealth technology. Many veterans reported that TelePain video and audio quality was *“good”* or *“fine.”* When asked to describe the video and sound quality during their visit, one veteran said: “*It’s been really good*.” Another veteran responded, “*It was fine. No problems at all*.” Another veteran said, “*The audio was good. I could hear them; they could hear us*.” One veteran suggested that their experience with technology was positive because the VA has improved telehealth technology access over time: “*I think that the VA has pretty much nailed it with the ease of access now. I would say prior experience tells me that they’ve really gotten all of the bugs out of the system.*”

Those who did have technological issues reported that the TelePain staff helped them navigate the problem. One patient said, “*Yeah, [TelePain] is helping me with the tablet and all of that other stuff, because I’m not really computer literate*.” Another veteran who had trouble with the audio on their computer said that they came up with a solution with their provider to fix the problem: *“She calls me. So that solved the problem. We both came up with [the idea] at the same time*.”

Several patients did have suggestions for telehealth technological improvements. One veteran who had participated in a telehealth group pain education class from their local CBOC said: *“It’d be nice if it were a little bit bigger TV, a little bigger. But we could hear fine. I’m not sure what kind of a monitor they have on us, if they even see us. There were a couple times, and it wasn’t always that way, but we were just forgotten.”* When asked about the quality of the video, another veteran responded, “*That I think could be improved. It wasn’t bad, I think it could just be improved with better monitors, or maybe a better camera system*.”

While some patients had suggestions for technological improvement, a majority had no significant problems using telehealth technology. Patients who did have minor technological problems were able to collaborate with TelePain providers and staff to find a solution.

## Discussion

In this qualitative evaluation of rural veterans’ experiences using a telehealth-based program for chronic pain management, patients reported positive experiences and general satisfaction across several domains: experiences with TelePain providers, gaining information and resources for pain care, convenience of TelePain, and technology usability. While most patients were satisfied with the program, some patients reported experiencing barriers that affected their participation in telehealth pain care: lack of program follow-up, negative perceptions of mental health pain care, and preference for in-person treatment. This study presents patient experiences with TelePain in order to further understand the issues that affect rural veterans’ participation in care and improve remote pain care services for this population.

Overall, patients reported positive experiences using TelePain and a majority reported that they would consider using it in the future. These findings align with previous research that has reported high rates of patient satisfaction with telehealth [7, 20–22] and found that improved access to care was associated with patient satisfaction [20]. Many patients described their provider as kind and/or knowledgeable, echoing previous research reporting telehealth provider behaviors that facilitate patient satisfaction include professionalism, rapport with patient, strong communication, empathy, shared decision making, and the ability to listen [23–26]. Patients also found that TelePain providers helped them manage their chronic pain by providing valuable information and resources. In addition, patients appreciated the convenience of a telehealth program that helped them alleviate their travel burden for specialty services. Convenience and perceived quality of care have shown to be important to telehealth patients [20], and distance to healthcare services is a known barrier to accessing quality care for rural veterans [27]. These findings align with previous research that found that telehealth decreased travel burden for patients [28] and that convenience is one of the biggest contributing factors to patient satisfaction with telehealth [29].

Patients reported that a perceived lack of TelePain follow-up limited their ability to participate in the program. These experiences included not receiving expected follow-up calls from TelePain providers or difficulty scheduling appointments. Previous research has identified organizational challenges such as clinical workflow issues and administrative burden as barriers to telehealth utilization [30, 31]. Ongoing quality improvement evaluations in the TelePain clinic have identified a workflow issue in the referral process, namely that referring providers often do not include necessary risk information that the program uses to triage all referrals (namely, assessment of suicide risk to ensure appropriate and safe care matched to the patient’s current risk level). When considering hub-and spoke specialty programs for rural patients, quality improvement efforts should focus on streamlining the referral process and educating referring providers on what is needed for a successful referral. While these backchannel, provider-to-provider communication gaps might not need to be explained fully to patients, the absence of any explanation or contact created a negative experience for some veterans. This finding indicates that improved care coordination is needed to improve patient experience of TelePain.

When asked if there were any factors that influenced their decision to participate in TelePain, some patients expressed negative perceptions of mental health pain care and questioned whether such treatment was effective for pain. Extensive research has previously examined the stigma of seeking mental health treatment among the veteran population, [32–34] which is associated with decreased likelihood of mental health utilization and greater perceived barriers to care [35]. Previous research has identified stigma, vulnerability, and a lack of trust as barriers to VA mental health services use [36]. One study found that “therapy-related barriers,” such as doubts about the effectiveness or success of treatment, contributed to veterans’ negative perceptions of VA behavioral pain providers and an unwillingness to seek mental health care [37]. Although a majority of patients had positive experiences with their TelePain providers, it is important to acknowledge that mental health stigma negatively impacts perceptions of mental health pain providers and treatments that address the psychosocial aspects of chronic pain. While telehealth reduces or removes many logistical barriers to accessing psychosocial pain care [38], interventions that enhance motivation to engage in psychosocial pain treatments are also needed [39].

Several patients reported hesitations to use telehealth in place of in-person appointments because they preferred face-to-face meetings with their providers. In a previous study examining patient satisfaction with a telemedicine pain clinic program, lowest mean satisfaction scores were reported when participants were asked to compare the care they received by telehealth to an in-person visit [40], indicating that some patients did not view telehealth as a substitute for in-person meetings. In other studies where a majority of patients were satisfied with telehealth services, a small number of patients still preferred face-to-face consultations [41, 42], suggesting that there are some patients who do not find telehealth comparable to in-person visits. Additional research is needed to further investigate patient preferences for in-person care relative to telehealth pain care and clinical outcomes.

Although a minority of patients suggested that VA’s telehealth technology could be improved, most patients did not have any significant problems using telehealth technology and reported general satisfaction with tech usability. This finding aligns with previous studies that have reported high levels of patient satisfaction with audio-visual quality of telehealth [25, 43] and usability of telehealth technology [44, 45]. When patients experienced minor problems using telehealth technology, they were able to collaborate with TelePain staff and providers to resolve these issues. This finding suggests that telehealth technology itself is not likely to be a barrier for veterans, and that VISN 20 TelePain may serve as an example for other VA pain programs implementing video telehealth services.

## Limitations

First, our sample was restricted to a predominantly white and male veteran population, thus limiting our ability to draw inferences about the acceptability of TelePain for a more diverse patient group. Further, patient race and sex were extracted from the electronic health record rather than self-reported, so participants may be misclassified [46, 47]. Second, a majority of our sample (*n* = 11) had participated in TelePain, as opposed to 5 veterans who had not. Therefore, this sample may not reflect all the challenges or barriers patients encountered that may have limited TelePain accessibility. Third, our methodology consisted of one interview with patients without follow-up assessments; consequently, these findings may not reflect changes in patients’ experiences after continued participation in TelePain. Fourth, because recruitment was conducted from a list of patients referred to TelePain, study staff were not aware of which participants did or did not participate in TelePain until they discussed their experiences during the interview. Therefore, we could not stratify recruitment by participation in the program, so there were not equal numbers of patients who did and did not participate.

## Conclusions

In this sample of rural veterans who used TelePain, many reported positive experiences with TelePain providers and general satisfaction with the program. Patients valued having supportive, knowledgeable providers who provided them with useful information and resources. In addition, veterans appreciated the convenience that telehealth visits offered. Barriers to care included perceived lack of TelePain follow-up, negative perceptions of mental health pain care, and preferences for in-person treatment. These results suggest that while a majority of veterans were satisfied with the program, streamlined referral and scheduling processes and interventions to enhance patient motivation for psychological pain care are needed to improve the implementation of pain telehealth. Although some patients reported that telehealth video and audio could be improved, most patients did not have any significant problems using the technology, indicating that the use of TelePain is feasible for rural veterans. The experiences of these veterans illustrate how TelePain facilitated access to pain care and helped them manage chronic pain, suggesting that the delivery of pain services over video telehealth technology is an acceptable modality for this patient population.

## Supplementary information

**Additional file 1**

**Additional file 2**

## Data Availability

The datasets used and/or analyzed during the current study are available from the corresponding author on reasonable request.
