# Supplementary material for: “There’s a huge benefit just to know that someone cares:” a qualitative examination of rural veterans’ experiences with TelePain
Source: BMC Health Serv Res. 2021 Oct 16;21:1111. doi: 10.1186/s12913-021-07133-5 (PMC8520618; doi:10.1186/s12913-021-07133-5)
Supplement: Supplementary file 1 — Additional file 1 [file 12913_2021_7133_MOESM1_ESM.docx]

**Patient Interviews**

**VISN 20 TelePain Patients – Individual Interview Guide, Patients who did NOT use TelePain**

**Section 1: Not connecting with TelePain**

1. Just to get started, tell me, how do you currently manage your chronic pain?
   1. How satisfied are you with your current pain management?
   2. Have you seen someone for Pain or used a Pain Clinic at VA?
   3. How is your pain care currently being managed through the VA?

*Our records indicate you were referred to the TelePain program by your provider [insert name] on [date]. Does that sound familiar?*

*Description, if needed: TelePain is a telehealth program that involves either going to a clinic or using technology in your house to meet with a pain specialist by video. That is, the pain specialist is located in the Seattle area, and your visit is done using the computer or television. Due to COVID, the meeting could also take place over the phone.*

***IF YES:***

1. How did you first hear about TelePain? What were you told about TelePain?
2. Walk me through what happened next after your provider referred you.
   1. What did you expect to happen next?

*Our records indicate that you did not meet with a TelePain provider [or, did not attend the class]. Is that correct?*

1. Can you tell me more about that?
2. What, if anything, got in the way of you seeing a TelePain provider?

**IF NO:**

1. How does seeing a pain specialist over video telehealth fit or not fit in to your chronic pain care?
2. What, if anything, would make you want to see a pain specialist using telehealth?
   1. If needed: Is there anything that could be changed about the TelePain program to make it a better fit for you?
3. How can your VA providers or the VA help you get the treatment you need for chronic pain?

**Section 2: Wrap-up**

1. Is there anything regarding your chronic pain care that we haven’t talked about that you would like to discuss?

- *Safety valve question****:*** What do you want us to know about TelePain to improve the program for Veterans like you?

*Thank you very much for taking the time to participate in this interview. Your responses have been very helpful and will help us improve care for Veterans with chronic pain.*
