# Supplementary material for: “There’s a huge benefit just to know that someone cares:” a qualitative examination of rural veterans’ experiences with TelePain
Source: BMC Health Serv Res. 2021 Oct 16;21:1111. doi: 10.1186/s12913-021-07133-5 (PMC8520618; doi:10.1186/s12913-021-07133-5)
Supplement: Supplementary file 2 — Additional file 2 [file 12913_2021_7133_MOESM2_ESM.docx]

**Patient Interview Guide**

**VISN 20 TelePain Patients – Individual Interview Guide, Patients who did use TelePain**

**Section 1: Connecting with TelePain**

1. Just to get started, tell me, how do you currently manage your chronic pain?

Our records indicate that you saw a TelePain provider [insert name] on [date] to discuss pain treatment options. Does that sound familiar?

*Description, if needed: TelePain is a telehealth program that involves either going to a clinic or using technology in your house to meet with a pain specialist by video. That is, the pain specialist is located in the Seattle area, and your visit is done using the computer or television. Due to COVID, the meeting could also take place over the phone.*

1. How did you find out about TelePain?
2. What were you told about TelePain?
3. Tell me about getting scheduled for or set up with your session/class.
   1. What worked well? What could have been improved?
4. Tell me about seeing a TelePain provider [Or, tell me about attending the TelePain class].
   1. What, if anything, did you like?
   2. What, if anything, didn’t you like?

If needed *(optional probes):*

- 1. Tell me about what information was covered in the session or class.
     1. What was useful or helpful? What was not useful or helpful?
     2. *If multiple appointments:* Tell me about your first session with a TelePain provider…
  2. Tell me about the format of the session or class.
     1. What worked well? What could have been improved?
  3. What, if anything, were you expecting from TelePain?
     1. Did the session/class meet your expectations?

**Section 2: Outcomes**

1. Did TelePain help you to manage your chronic pain?
   1. Why or why not?
   2. Did anything about your pain management change as a result of your visit/class?
2. What would you change, if anything, about TelePain?
3. What are the next steps for your pain care? [if needed, what happens next for your pain care?]
   1. Would you consider seeing a TelePain provider over telehealth (again / in the future)?

**Section 3: Usability of TelePain technology**

*Now we’d like to ask you some questions about what it was like to see a TelePain provider using telehealth technology.*

1. To clarify, how did you connect with the provider?
   1. Was it over the phone, through video conference, did you go to a clinic?
2. When you saw a TelePain provider…
   1. Did you have any problems with knowing when and where your appointment was?
   2. Did you have any problems with the set-up of the video?
   3. How was the video quality during your TelePain visit?
   4. How was the sound quality during your TelePain visit?
   5. Do you have any suggestions to improve your experience with the technology?

**Section 4: Wrap-up**

1. Is there anything regarding TelePain that we haven’t talked about today that you would like to discuss?

- *Safety valve question****:*** What do you want us to know about TelePain to improve the program for Veterans like you?

*Thank you very much for taking the time to participate in this interview. Your responses have been very helpful and will help us improve care for Veterans with chronic pain.*
